# ﻿*Nanhuaphasma* Chen, He & Li, 2002 is a junior synonym of *Dajaca* Brunner von Wattenwyl, 1893 (Phasmatodea, Aschiphasmatidae, Dajacini)

**DOI:** 10.3897/zookeys.1082.73272

**Published:** 2022-01-18

**Authors:** Chong-Xin Xie, Jun WenYu-Han Qian1

**Affiliations:** 1 Key Laboratory for Forest Resources Conservation and Utilization in the Southwest Mountains of China, Ministry of Education, Southwest Forestry University, Kunming, Yunnan 650224, China Southwest Forestry University Kunming China

**Keywords:** External morphology, new synonym, stick insects, taxonomy

## Abstract

The genus *Nanhuaphasma* Chen, He & Li, 2002 was established as a member of the family Pseudophasmatidae Rehn, 1904 (now belonging to Aschiphasmatidae Brunner von Wattenwyl, 1893) based on the male of *N.hamicercum* Chen & He, 2002. We review the status of *Nanhuaphasma* and *N.hamicercum* by examining the holotype and male and female non-types which were collected in same location as the holotype. We find that *Nanhuaphasma* is a junior synonym of *Dajaca* Brunner von Wattenwyl, 1893 and *N.hamicercum* is a junior synonym of *D.napolovi* Brock, 2000. Complementing egg morphology of *D.napolovi* and keys to eight species of *Dajaca* are provided.

## ﻿Introduction

*Nanhuaphasma* was established by Chen, He & Li in 2002, as a genus of the subfamily Pseudophasmatinae Rehn, 1904 and the family Pseudophasmatidae Rehn, 1904. This genus only includes *Nanhuaphasmahamicercum* Chen & He, 2002, with its holotype collected on Jianfengling Mountain in Hainan Province of China, and the male paratype collected on Mount Daqing in Guangxi Province of China; its female was unknown in the original description. From the original description, the genus has the following characteristics: body medium-sized, without spines or granules, and covered with dense, short, yellow villi. Antennae filiform, distinctly segmented, longer than apex of fore legs. Pronotum wider than length and anterior with a pair of elliptically cavities, median segment longer than metanotum. Fore femora short and slightly curved, without distinct carina, mid and hind tibiae without spines or denticles, undersides with triangular cavities apically, tarsi V segmented, ungues not pectinate. Based on the above characteristics, Hennemann et al. (2008) thought *Nanhuaphasma* might belong to Aschiphasmatini Brunner von Wattenwyl, 1893. Ho (2016) considered *Nanhuaphasma* to belong to Dajacini Bragg, 2001.

The Dajacini are similar to Aschiphasmatini, but they are distinguished only by the ungules which are not pectinate (Bragg 2001). *Dajaca* is the type genus of Dajacini and only eight species worldwide are known; they have have been described from Indonesia, Malaysia, Brunei, Vietnam, Myanmar, and China (Brock et al. 2021). Bragg (2001) revised *Dajaca* and provided an identification key. Zompro (2004) revised *Phaeophasma* as a junior synonym of *Dajaca*. Seow-Choen (1998, 2016, 2017, 2020) systematically worked through *Dajaca* based on specimens from Borneo and Sumatra.

We observe that *N.hamicercum* is similar to species of *Dajaca* based on diagnostic features of the holotype and new specimens which were collected at the same location as the holotype. Here, we resolve the status of *Nanhuaphasma* and conclude that it is a junior synonym of *Dajaca*. We also provide new keys to *Dajaca* based on external morphology. Considering the individual variability of *D.napolovi*, we redescribe the female and male. The egg of *D.napolovi* is described for the first time in this paper.

## ﻿Materials and methods

The recently collected specimens include 2♂, 2♀, and 3 eggs of *Nanhuaphasmahamicercum* collected from Jianfengling National Forest Park in Hainan Province, China. These specimens are pinned and deposited in the Insect Collection of Southwest Forestry University (**SWFU**), Yunnan Province, China. The holotype and paratype of *N.hamicercum* deposited in the Institute of Zoology, Chinese Academy of Sciences (**IZCAS**), Beijing, China. Retrieved from Phasmida Species File (Brock et al. 2021), the holotype and paratype of *D.napolovi* deposited in Natural History Museum, London, England (**NHMUK**), were photographed by Paul Brock, and the images are under copyright to the Natural History Museum, London.

Morphological observations were made with a SOPTOP SZ stereomicroscope (Sunny Group Co., Ltd, China). Digital images were obtained using a Liyang Super Resolution System LY-WN-YH (Chengdu Liyang Precision Machinery Co., Ltd, China). Whole view images of the new specimens were taken with Canon 5ds digital camera and LAOWA 100 mm F2.8 2× macro lens (Anhui Changgeng Optics Technology Co., Ltd, China). Image Stacking was done using Zerene Stacker (Zerene Systems LLC, USA). Morphological terminology follows that of Bragg (1997, 2001) and Vallotto et al. (2016).

## ﻿Taxonomic account

### ﻿Genus Dajaca

Taxon classificationAnimaliaPhasmatodeaAschiphasmatidae

Brunner von Wattenwyl, 1893

360E827C-F750-5004-9DC3-54FFFCD02EB0

Dajaca Brunner von Wattenwyl, 1893: 99 (original description; type species: Dajacamonilicornis Redtenbacher, 1906; type locality: Tam Dao, 55 km NNW Hanoi, Vietnam).
Nanhuaphasma syn. nov. Chen, He & Li, 2002: 100 (original description; type species: Nanhuaphasmahamicercum Chen & He, 2002; type locality: Jianfengling National Forest Park, Hainan province, China); Chen and He 2008: 365 (redescription).

#### Remarks.

Head flattened, antennae long. Median segment twice as long as metanotum. Ungues not pectinate. Male apterous or winged, females apterous. Legs short, femora laterally compressed, dorsal surface rounded; ventral carinae with only a few minute spines or unarmed. Fore femora more or less straight. Tibiae unarmed (Bragg 2001; Zompro 2004). After comparing the diagnostic features, *Nanhuaphasma* shows similar characters to the *Dajaca*, and we could not find significant morphological differences between the two and therefore consider *Nanhuaphasma* to be a junior synonym of *Dajaca*.

For the convenience of research, we hereby give the Chinese name. Latin *Dajaca* in Chinese, transliterated as “达伽卡”, simplified as “达”.

### ﻿ Dajaca napolovi

Taxon classificationAnimaliaPhasmatodeaAschiphasmatidae

Brock, 2000

D59A9693-8A75-53D9-88B1-5F4865283408

**Figure 1.**
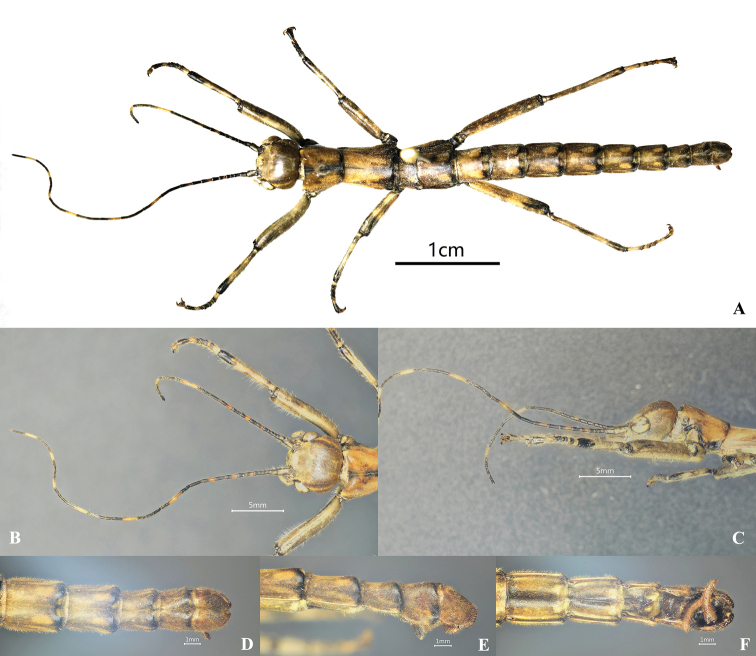
*Dajacanapolovi*, male, non-type (collected from Jianfengling National Forest Park in Hainan Province, China). **A** habitus, dorsal view **B** head, dorsal view **C** head, lateral view **D** terminalia, dorsal view **E** terminalia, lateral view **F** terminalia, ventral view.

**Figure 2.**
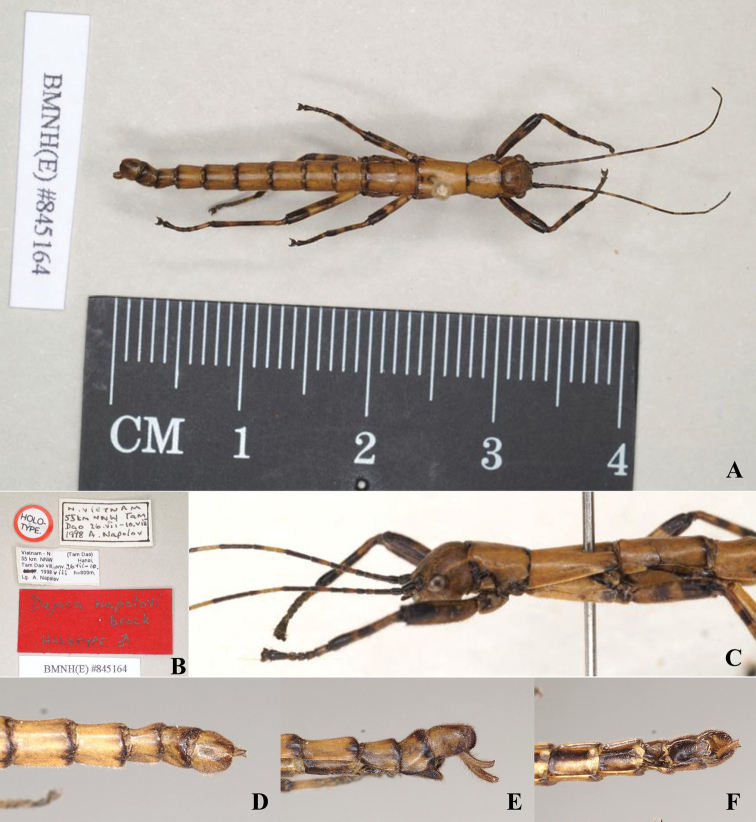
*Dajacanapolovi*, male, holotype (from Phasmida Species File 2021, photos by Paul Brock; published under CC BC -ShareAlike 4.0 International License). **A** habitus, dorsal view **B** data labels **C** head, lateral view **D** terminalia, dorsal view **E** terminalia, lateral view **F** terminalia, ventral view.

**Figure 3.**
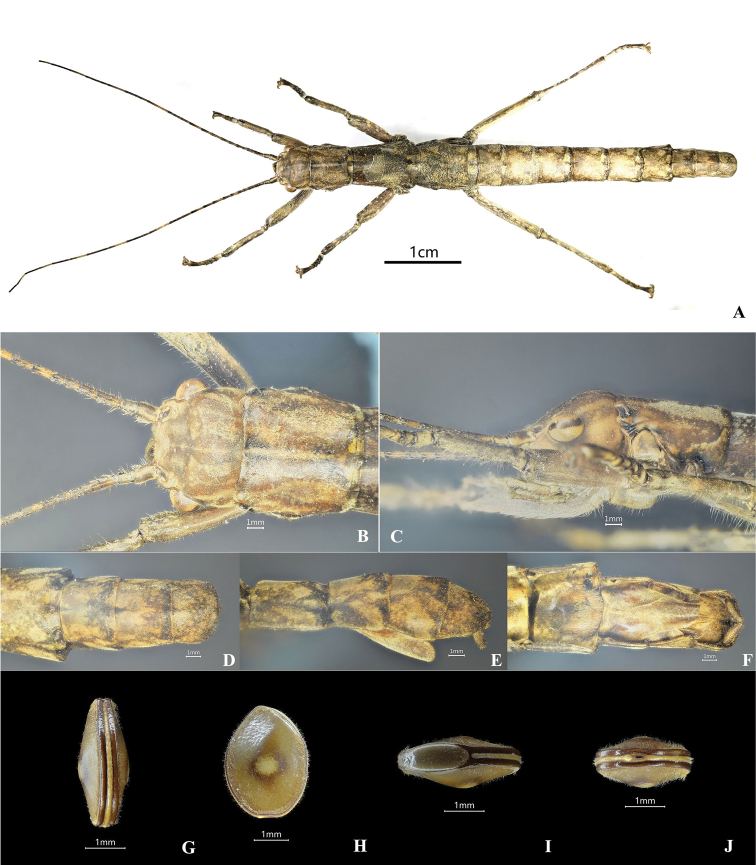
*Dajacanapolovi*, female & egg, non-type (collected from Jianfengling National Forest Park in Hainan Province, China). **A** female habitus, dorsal view **B** female head, dorsal view **C** female head, lateral view **D** female terminalia, dorsal view **E** female terminalia, lateral view **F** female terminalia, ventral view **G** egg, dorsal view **H** egg, lateral view **I** egg, opercular view **J** egg, polar view.

**Figure 4.**
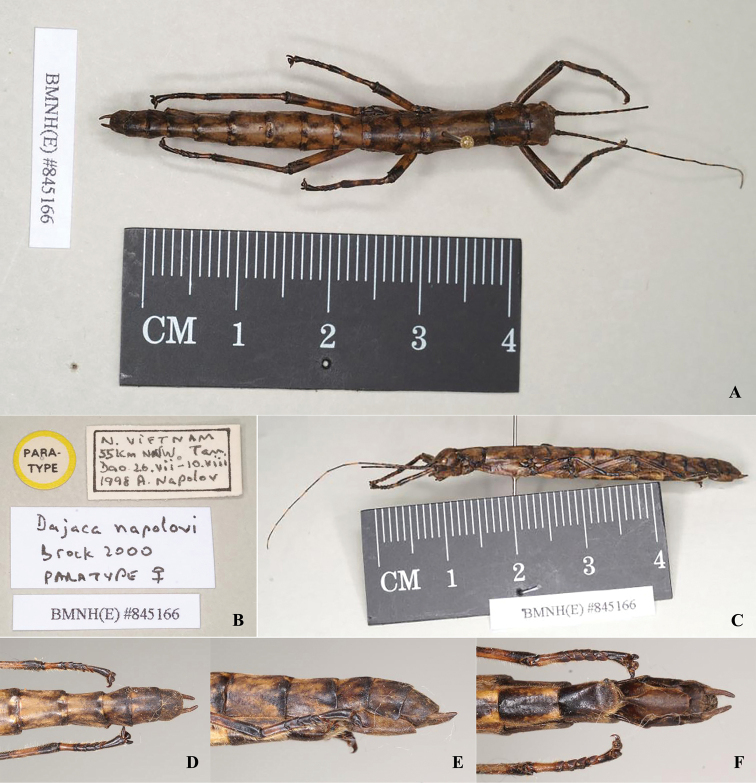
*Dajacanapolovi*, female, paratype (from Phasmida Species File 2021, photos by Paul Brock, published under CC BC -ShareAlike 4.0 International License). **A** habitus, dorsal view **B** data labels **C** habitus, lateral view **D** terminalia, dorsal view **E** terminalia, lateral view **F** terminalia, ventral view.

**Figure 5.**
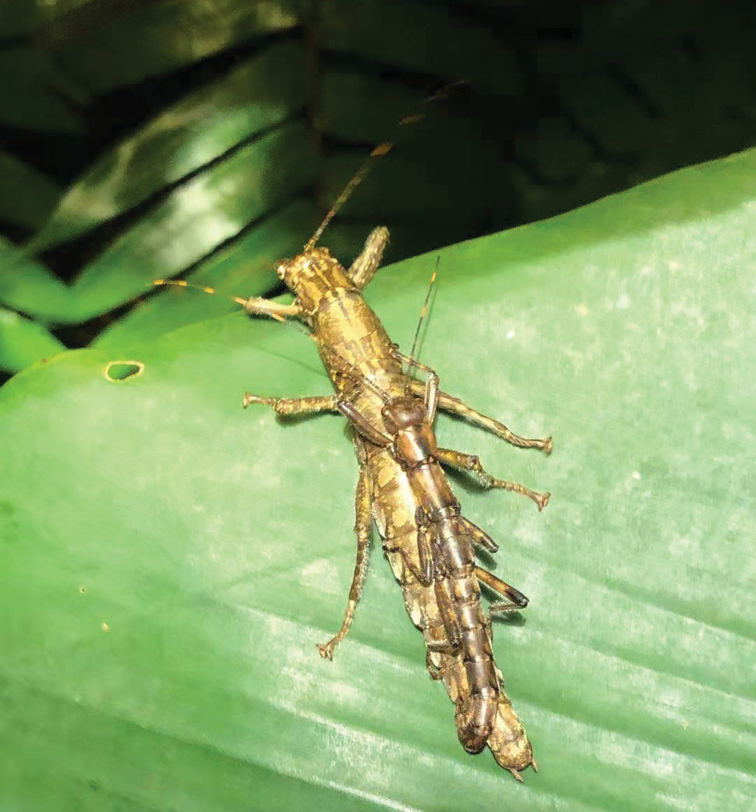
*Dajacanapolovi*, female and male mating in the wild (from Jianfengling National Forest Park in Hainan Province, China, photograph by Mr Yun-Hu Mo)

纳氏达䗛 Figures 1 , 2 , 3 , 4 , 5

Dajaca napolovi Brock, 2000: 2 (original description; type locality: Tam Dao, 55 km NNW Hanoi, Vietnam); Vallotto et al. 2016: 376; (described both male and female).
Nanhuaphasma hamicercum syn. nov. Chen & He, 2002: 100 (original description; type locality: Jianfengling National Forest Park in Hainan province, China; described male); Chen and He 2008: 365, 458 (redescription).

#### Material examined.

2♂, 2♀ and 3 eggs of *D.napolovi*, Jianfengling National Forest Park in Hainan Province, China, 18°44'35"N, 108°50'17"E, 1134 m alt., 6.VIII.2020, leg. Yun-Hu Mo; No. HN-25.

#### Description.

**Male.** Wingless, the general coloration of the body is yellowish brown, with a few dark brown or black markings and pale yellow pilosity (Figs 1A, 2A). ***Head.*** Smooth, approximately as long as pronotum; nearly square, length almost as long as broad, vertex humped. Antennae filiform, longer than forelegs, with yellow bands; scapus rectangular and flattened, longer than pedicellus, pedicellus cylindrical and slightly wider than the third segment. Eyes rounded, colored yellow with a black median line, occupying 1/2 of gena (Figs 1B, C, 2C). ***Thorax.*** Smooth and unarmed. Pronotum rectangular, longer than wide, gradually narrowed posteriorly. Mesonotum slender and parallel-sided, ca 1.3× as long as pronotum. Metanotum wider than long. Median segment as long as wide, 2× length of metanotum (Figs 1A, 2A, C). ***Abdomen.*** Cylindrical, smooth, lacking armature. Terga II–IX gradually narrowed. Anal segment with small notch in middle of posterior margin. Poculum flat and short, nearly reaching posterior margin of tergum IX, apex rounded. Cerci cylindrical, moderately long, and slightly incurving, apices with tiny spines (Figs 1A, D–F, 2A, D–F). ***Legs.*** Brown with irregular black stripes; all femora laterally compressed, more or less triangular, lacking dorsal carinae, ventral carinae distinct. Ventroanterior carina of prefemur with some minute spines, remainder unarmed (Figs 1A, 2A).

**Female.** Larger than male, general coloration of body dark to light brown, with a few dark brown or black markings and pale yellow pilosity (Figs 3A, 4A, C). ***Head.*** Smooth, shorter than pronotum; rectangular, wider than long, vertex slightly humped. Antennae filiform, longer than forelegs, with yellow bands; scapus rectangular and flattened, longer than pedicellus, pedicellus cylindrical and slightly wider than third segment. Eyes rounded, colored yellow with a black median line, occupying 1/2 of gena (Figs 3B, C, 4A, C). ***Thorax.*** Smooth and unarmed. Pronotum somewhat square, length almost as long as broad. Mesonotum anteriorly slightly narrowed and gradually broadening posteriorly, ca 1.5× as long as pronotum. Metanotum wider than long. Median segment slightly wider than long, 2× length of metanotum (Figs 3A, 4A, C). ***Abdomen.*** Cylindrical, smooth, and lacking armature. Terga II–VII slightly broad, tergum VIII–IX distinctly narrowed. Anal segment as wide as tergum IX, posterior margin broadly rounded. Sternum VII lacking praeopercular organ. Lamina subgenitalis relatively long, without carinae, extending to posterior of tergum IX, anterior broad and posterior gradually narrowed, apex rounded and almost covering the ovipositor completely, paraprocts and epiproct not covered by lamina subgenitalis. Cerci cylindrical, moderately long, and slightly incurved, apices without tiny spines (Fig. 3A, D–F, 4A, C, D–F). ***Legs.*** Brown with irregular black stripes, all femora laterally compressed, more or less triangular, lacking dorsal carinae, ventral carinae distinct (Figs 3A, 4A, C).

**Eggs.** Capsule a laterally flattened disk and slightly swollen in center; capsule longer than high, uniformly mid-brown, densely setose; rim of operculum and micropylar plate dark brown. Operculum elongate-oval, lacking capitulum. Micropylar plate a narrow band which extends around rim of egg, starting and ending at operculum. Micropyle situated at end of polar; micropylar plate slightly wider at this point (Fig. 3G–J).

#### Measurements (mm).

**Male.** Body length 41–47; head length 5.0–5.5; pronotum length 3.5–4.0; mesonotum 5.3–5.5; metanotum 1.5–2.0; median segment 3.5–4.0; profemora 7.0–8.0; mesofemora 6.0–7.0; metafemora 9.0–10.0; protibiae 5.5–6.0; mesotibiae 5.0–5.5; metatibiae 8.5–9.0. **Female.** Body length 58–61; head length 5.7–6.0; pronotum length 7.5–8.0; mesonotum 8.0–9.0; metanotum 2.6–3.0; median segment 4.5–5.0; profemora 7.5–9.0; mesofemora 7.0–8.0; metafemora 11.0–12.0; protibiae 6.5–7.0; mesotibiae 6.0–6.5; metatibiae 9.5–10.0. **Egg.** Width 1.2–1.3; height 2.4–2.6; length 3.0–3.3.

#### Remarks.

Comparing the descriptions and illustrations in the original texts, the holotypes and the new specimens collected from the type locality, we find that *Nanhuaphasmahamicercum* shows similar characters to *Dajacanapolovi*, such as being wingless and having the body smooth and unarmed. Male anal segment with a small notch in the middle of the posterior margin; poculum flat and short, nearly reaching to the posterior margin of tergum 9, apex rounded; cerci slightly incurving, apices with tiny spines. Female anal segment posterior margin broadly rounded; sternum 7 lacking preopercular organ; lamina subgenitalis relatively long, without carinae; anterior broadly and posterior gradually narrowed, apex rounded; almost covering the ovipositor completely, paraprocts and epiproct not covered by lamina subgenitalis. After the above comparison, we could not find significant differences between the two species and therefore consider *N.hamicercum* as a junior synonym of *D.napolovi*. Considering the geographical and intraspecific variability of *D.napolovi*, the colors of body are slightly different; due to contraction of the abdomen segments, the lamina subgenitalis sometimes extends slightly to the posterior of tergum IX, sometimes distinctly surpassing it to reach the posterior of tergum IX.

##### ﻿List of the species and distributions of *Dajaca*

*D.alata* (Redtenbacher, 1906) [Malaysia]

*D.chani* Seow-Choen, 1998 [Malaysia]

*D.filiformis* Bragg, 1992 [Malaysia]

*D.monilicornis* Redtenbacher, 1906 [Malaysia]

*D.napolovi* Brock, 2000 [China: Hainan, Hong Kong, Guangxi; Vietnam] = *Nanhuaphasmahamicercum* Chen & He, 2002, syn. nov.

*D.nigrolineata* Hennemann, Conle & Bruckner, 1996 [Myanmar]

*D.swiae* Seow-Choen, 2020 [Indonesia: Sumatra]

*D.viridipennis* Bragg, 2001 [Indonesia: Sarawak]

### ﻿Key to males of *Dajaca* worldwide

**Table:** 

1	Winged	**2**
–	Wingless	**5**
2	Wings reaching to ca 1/2 way along 7^th^ segment	***D.alata* (Redtenbacher, 1906)**
–	Wings not reaching to 1/2 way along 7^th^ segment	**3**
3	Wings not reaching end of 5^th^ segment	***D.monilicornis* Redtenbacher, 1906**
–	Wings reaching to ca 1/2 way along 6^th^ segment	**4**
4	Body green, ventral surface of femora and tibiae black	***D.viridipennis* Bragg, 2001**
–	Body and legs brown	***D.filiformis* Bragg, 1992**
5	Body with a distinct black median line	***D.nigrolineata* Hennemann, Conle & Bruckner, 1996**
–	Body without median line	**6**
6	Body and legs green; antenna with yellow band	***D.chani* Seow-Choen, 1998**
–	Body and legs brown and a few black stripes; antennal without yellow band	***D.napolovi* Brock, 2000**

### ﻿Key to females of *Dajaca* worldwide

**Table:** 

1	Body with a distinct black median line	***D.nigrolineata* Hennemann, Conle & Bruckner, 1996**
–	Body without a median line	**2**
2	Hind legs reaching or surpassing to 8^th^ segment	**3**
–	Hind legs not surpassing to 8^th^ segment	**4**
3	Body and legs green; body length <45 mm	***D.chani* Seow-Choen, 1998**
–	Body and legs brown and with a few black stripes; body length >45 mm	***D.napolovi* Brock, 2000**
4	Antennal segments 3–5 swollen; body and legs green	***D.monilicornis* Redtenbacher, 1906**
–	Antennal segments 3–5 slender; body and legs brown	**5**
5	Ventro-anterior carina of metafemora with 4 small teeth	***D.filiformis* Bragg, 1992**
–	Ventro-anterior carina of metafemora with 5 teeth and ventro-anterior carina bearing 3 or 4 teeth	***D.swiae* Seow-Choen, 2020**

## ﻿Conclusions

Hainan Province is the largest tropical island in China, where the phasmids are a priority for biodiversity conservation. *Nanhuaphasmahamicercum* was collected in Jianfengling National Forest Park by Mr Yun-Hu Mo who photographed a mating pair of *N.hamicercum* (Fig. 5). Comparing the original description and the diagnostic features of the holotype, paratype and non-type specimens, we found that *Nanhuaphasma* should be a junior synonym of *Dajaca* and *N.hamicercum* should be a junior synonym of *D.napolovi*.

## Supplementary Material

XML Treatment for Dajaca

XML Treatment for Dajaca napolovi
